# In the September 2013 issue

**DOI:** 10.1590/S1980-57642013DN70300001

**Published:** 2013

**Authors:** Cássio M.C. Bottino, Jerson Laks

For this issue of **Dementia** *&* **Neuropsychologia**, we were invited to present an account of the present knowledge on neuropsychiatric symptoms of dementia. This area of research was neglected for many years but, in the last decade, clinicians and researchers have started to recognize the pathophysiology of these symptoms and their importance for the management of patients with dementia.

Since 2002, papers have described the prevalence of dementia in the population aged over 65 years in Brazil. The first such study reported a dementia prevalence of 7.1%^1^ in older subjects living in Catanduva, São Paulo state. Another study^2^ estimated the prevalence of dementia in São Paulo city as 12.9%, in the population aged over 60 years, figures equal to or higher than the prevalence rates of dementia found in developed countries^3^.

In parallel with the interest in dementia, the Behavioral and Psychological Symptoms in Dementia (BPSD) or neuropsychiatric symptoms in dementia, have been studied more intensively in the last few years. On a PUBMED search using the key words BPSD or neuropsychiatric symptoms, dementia, and Brazil, 32 original articles and reviews published between January 1999 and June 2013^4^ were retrieved. The articles found in PUBMED showed the field in Brazil is moving ahead, with a shift from initial reports of prevalence of symptoms to more recent studies focusing on the neurobiology and non-pharmacological treatment of these symptoms.

For this issue of the journal, the authors have presented an interesting overview of the current research in the area. Two reviews which update knowledge on the instruments used to assess and rate neuropsychiatric symptoms and on the neurobiology of neuropsychiatric symptoms and neuroimaging associated with them, sets the scene for the original contributions. The original contributions cover the wide field related to neuropsychiatric symptoms in dementia. This edition carries articles on new scale validations, neuropsychiatric features of special symptoms, issues concerning biomarkers of neuropsychiatric symptoms, and caregiver burden and stress. Last but not least, an intriguing case report provides further information which may be discussed and contributes to the readers' knowledge. We are delighted to have participated in this initiative by Dementia & Neuropsychologia to provide readers and research colleagues with such an important contribution. Also, we would like to thank all the authors for their excellent manuscripts.

Cássio M.C. BottinoJerson Laks
